# Davey-Stewartson Equation with Fractional Coordinate Derivatives

**DOI:** 10.1155/2013/941645

**Published:** 2013-11-25

**Authors:** H. Jafari, K. Sayevand, Yasir Khan, M. Nazari

**Affiliations:** ^1^Department of Mathematics and Computer Science, University of Mazandaran, P.O. Box 47416-1467, Babolsar, Iran; ^2^International Institute for Symmetry Analysis and Mathematical Modelling, Department of Mathematical Sciences, North-West University, Mafikeng Campus, Private Bag X 2046, Mmabatho 2735, South Africa; ^3^Faculty of Mathematical Sciences, Malayer University, P.O. Box 65719-95863, Malayer, Iran; ^4^Department of Mathematics, Zhejiang University, Hangzhou 310027, China

## Abstract

We have used the homotopy analysis method (HAM) to obtain solution of Davey-Stewartson equations of fractional order. The fractional derivative is described in the Caputo sense. The results obtained by this method have been compared with the exact solutions. Stability and convergence of the proposed approach is investigated. The effects of fractional derivatives for the systems under consideration are discussed. Furthermore, comparisons indicate that there is a very good agreement between the solutions of homotopy analysis method and the exact solutions in terms of accuracy.

## 1. Introduction

In recent years, fractional differential equations (FDEs) have been the focus of many studies due to their appearance in various fields such as physics, chemistry, and engineering [1–7]. On the other hand, much attention has been paid to the solutions of fractional differential equations. Several techniques including Adomian decomposition method (ADM) [8, 9], Laplace decomposition method [10], homotopy perturbation method (HPM) [11], variational iteration method (VIM) [11], and differential transform method [12] have been used for solving a wide range of problems. Another powerful analytical method, called the homotopy analysis method (HAM), was first proposed by Liao in his Ph.D. thesis [13]. The HAM contains a certain auxiliary parameter *ℏ* which provides us with a simple way to adjust and control the convergence region and rate of convergence of the series solution. This method has been successfully applied to solve many types of nonlinear problems [14–17]. For instance, Jafari and Seifi have solved diffusion-wave equations and system of nonlinear fractional partial differential equations using homotopy analysis method [18, 19].

In this paper, the homotopy analysis method [13, 20] is applied to solve fractional Davey-Stewartson equations:

(1) 12σ4∂αq∂yα+12σ2∂2q∂x2+i∂q∂t+λ|q|2q−∂ϕ∂xq=0,∂2ϕ∂x2−σ2∂αϕ∂yα−2λ∂(|q|2)∂x=0, 1<α≤2, λ∈ℜ,  σ∈𝒞.

The special case {*α* = 2, *σ* = 1} is called the classical DS-I equation, while $\left\{\alpha =2,\sigma =\pm \sqrt{-1}\right\}$ is the classical DS-II equation. The parameter *λ* characterizes the focusing or defocusing case. The classical Davey-Stewartson I and II are two well-known examples of integrable equations in two space dimensions, which arise as higher dimensional generalizations of the nonlinear Schrödinger equation [21]. Although there are a lot of studies for the classical Davey-Stewartson equation and some profound results have been established, it seems that detailed studies of the fractional Davey-Stewartson equation are only beginning. We intend to apply the homotopy analysis method to solve the fractional Davey-Stewartson equations. We will also present numerical results to show the nature of the curves/surfaces as the fractional derivative parameter changed. 

## 2. Preliminaries and Notations

This section deals with some preliminaries and notations regarding fractional calculus. For more details see [6, 22–24].

**Definition 1**

A real function *u*(*t*), *t* > 0, is said to be in the space *C*_*α*_, *α* ∈ *ℜ*, if there exists a real number *p*(>*α*), such that *u*(*t*) = *t* ^*p*^ *u*_1_(*t*), where *u*_1_(*t*) ∈ *C*[0, *∞*), and it is said to be in the space *C*_*α*_^*m*^, *m* ∈ *𝒩*⋃ {0}, if and only if *u* ^(*m*)^(*t*) ∈ *C*_*α*_.

**Definition**

The (left sided) Riemann-Liouville fractional integral of order *α* > 0 of a function *u*(*t*) ∈ *C*_*α*_, *α* ≥ −1, is defined as

(2) $$\begin{array}{c} I_{t}^{\alpha }u\left(t\right)=\{\begin{array}{cc} \frac{1}{\Gamma \left(\alpha \right)}\int_{0}^{t}\frac{u\left(\tau \right)}{{\left(t-\tau \right)}^{1-\alpha }}d\tau , & \alpha >0,\;\;t>0,\\ u\left(t\right), & \end{array}\\ I_{t}^{\alpha }u\left(x,t\right)=\frac{1}{\Gamma \left(\alpha \right)}\int_{0}^{t}\frac{u\left(x,s\right)}{{\left(t-s\right)}^{1-\alpha }}ds,\;\alpha >0,\;\;t>0, \end{array}$$

where Γ(·) is the well-known Gamma function.

**Definition 3**

The (left sided) Riemann-Liouville fractional derivative of *u*(*t*), *u*(*t*) ∈ *C*_−1_^*m*^, *m* ∈ *𝒩* ⋃ {0}, of order *α* is defined as

(3) $$\begin{array}{c} D_{t}^{\alpha }u\left(t\right)=\frac{d^{m}}{dt^{m}}I_{t}^{m-\alpha }u\left(t\right),\;m-1<\alpha \leq m,\;\;m\in 𝒩. \end{array}$$

**Definition 4**

The (left sided) Caputo fractional derivative of *u*(*t*), *u*(*t*) ∈ *C*_−1_^*m*^, *m* ∈ *𝒩*⋃{0}, is defined as

(4) $$\begin{array}{c} D_{*t}^{\alpha }u\left(t\right)=\{\begin{array}{cc} \left[I_{t}^{m-\alpha }u^{\left(m\right)}\left(t\right)\right] & m-1<\alpha <m,\;\;m\in 𝒩,\\ \frac{d^{m}}{dt^{m}}u\left(t\right) & \alpha =m, \end{array}\\ D_{*t}^{\alpha }u\left(x,t\right)=I_{t}^{m-\alpha }\frac{\partial^{m}u\left(x,t\right)}{\partial t^{m}},\;m-1<\alpha <m,\\ D_{*t}^{\alpha }D_{t}^{m}u\left(t\right)=D_{*t}^{\alpha +m}u\left(t\right),\;m=0,1,\ldots ,n-1<\alpha <n. \end{array}$$

*Property.* Similar to integer-order differentiation, fractional differentiation is a linear operation:

(5) $$\begin{array}{c} D_{*t}^{\alpha }\left(\gamma u\left(t\right)+\eta v\left(t\right)\right)=\gamma D_{*t}^{\alpha }u\left(t\right)+\eta D_{*t}^{\alpha }v\left(t\right). \end{array}$$

### 2.1. The Relation between Fractional Derivative and Fractional Integral

**Theorem 5**

Assume that the continuous function *u*(*t*) has a fractional derivative of order *α*; then one has

(6) DtαItβu(t)={Itβ−αu(t)α<β,u(t)α=β,Dt−β+αu(t)α>β,ItαD∗tαu(t)=u(t)−∑k=0m−1u(k)(0+)tkk!,      m−1<α≤m, m∈𝒩,D∗tαItαu(t)={u(t),m−1<α≤m,m∈𝒩,ItαD∗tαu(t)+u(0),0<α<1.

## 3. Homotopy Analysis Method

Let us consider the following system of differential equations:

(7) $$\begin{array}{c} {𝒩}_{i}\left[u_{1}\left(x,y,t\right),\ldots ,u_{n}\left(x,y,t\right),x,y,t\right]=0,\;i=1,2,\ldots ,n, \end{array}$$

where *𝒩*_*i*_ are nonlinear operators and *u*_*i*_(*x*, *y*, *t*) are unknown functions. By means of generalizing the traditional homotopy method, Liao [15] constructed the so-called zero-order deformation equations:

(8) (1−p)ℒi[ϕi(x,y,t;p)−ui0(x,y,t)] =pℏi𝒩i[ϕ1(x,y,t;p),…,ϕn(x,y,t;p),x,y,t],                     i=1,2,…,n,

where *p* ∈ [0,1] is the embedding parameter, *ℏ*_*i*_ ≠ 0 are nonzero auxiliary parameters, and *ℒ*_*i*_ are auxiliary linear operators with the following property:

(9) $$\begin{array}{c} {ℒ}_{i}\left[c\right]=0,\;i=1,2,\ldots ,n, \end{array}$$

where *c* is constant. *u*_*i*0_(*x*, *y*, *t*) are initial guesses of *u*_*i*_(*x*, *y*, *t*), *ϕ*_*i*_(*x*, *y*, *t*; *p*) are unknown functions, respectively. It is important that one has great freedom to choose auxiliary things in HAM. Obviously, when *p* = 0 and *p* = 1, it holds

(10) ϕi(x,y,t;0)=ui0(x,y,t),ϕi(x,y,t;1)=ui(x,y,t),       i=1,2,…,n,

respectively. Thus, as *p* increases from 0 to 1, the solution *ϕ*_*i*_(*x*, *y*, *t*; *p*) varies from the initial guesses *u*_*i*0_(*x*, *y*, *t*) to the solution *u*_*i*_(*x*, *y*, *t*). Expanding *ϕ*_*i*_(*x*, *y*, *t*; *p*) in Taylor series with respect to *p*, we have

(11) ϕi(x,y,t;p)=ui0(x,y,t)+∑m=1+∞uim(x,y,t)pm,                  i=1,2,…,n,

where

(12) uim(x,y,t)=1m!∂mϕi(x,y,t;p)∂pm|p=0,               i=1,2,…,n.

If the auxiliary linear operators, the initial guesses, the auxiliary parameters *ℏ*_*i*_ are so properly chosen, the series (11) converges at *p* = 1; then we have

(13) uim(x,y,t)=ui0(x,y,t)+∑m=1+∞uim(x,y,t),                 i=1,2,…,n.

Define the vector

(14) u→im(x,y,t) ={ui0(x,y,t),ui1(x,y,t),…,uim(x,y,t)},         i=1,2,…,n, m=0,1,2,….

Differentiating (8) *m* times with respect to the embedding parameter *p*, then setting *p* = 0, and finally dividing them by *m*!, we obtain the *m*th-order deformation equations:

(15) ℒi[uim(x,y,t)−χmui m−1(x,y,t)] =ℏiRi,m(u→1 m−1(x,y,t),…,u→n m−1(x,y,t),x,y,t),                     m=1,2,…,

where

(16) Ri,m(u→1 m−1,…,u→n m−1,x,y,t) =1(m−1)!  ×∂m−1𝒩i[ϕ1(x,y,t;p),…,ϕn(x,y,t;p),x,y,t]∂pm−1|p=0,χm={0,  m⩽1,1,  m>1.  

The solution of the *m*th-order deformation equation (15) is readily found to be

(17) uim(x,y,t) =χmui m−1(x,y,t)+ℏiℒi−1   ×[Ri,m(u→1  m−1(x,y,t),…,u→n  m−1(x,y,t),x,y,t)],                    m=1,2,….

In this way, it is easy to obtain *u*_*im*_(*x*, *y*, *t*) for *m*⩾1, at *m*th-order; we have

(18) $$\begin{array}{c} u_{i}\left(x,y,t\right)=\sum_{m=0}^{M}u_{im}\left(x,y,t\right),\;i=1,2,\ldots ,n. \end{array}$$

When *M* → *∞*, we get an accurate approximation of the original equation (7).

## 4. Analysis of Fractional Davey-Stewartson Equations with the HAM

In this section we apply the proposed approach for solving the fractional Davey-Stewartson equations. Without loss of generality, first we separate the amplitude of a surface wave packet *q* into real part and imaginary part; that is, *q* = *u* + *iv*, (*i* ^2^ = −1). Then we rewrite the fractional Davey-Stewartson equations in the following form:

(19) ∂αu∂yα+1σ2∂2u∂x2−2σ4∂v∂t +2λσ4(u3+v2u)−2σ4(∂ϕ∂xu)=0,∂αv∂yα+1σ2∂2v∂x2+2σ4∂u∂t +2λσ4(v3+u2v)−2σ4(∂ϕ∂xv)=0,∂αϕ∂yα−1σ2∂2ϕ∂x2+2λσ2∂(u2+v2)∂x=0.

To solve (19) by means of homotopy analysis method, we choose the linear operators

(20) $$\begin{array}{c} {ℒ}_{1}={ℒ}_{2}={ℒ}_{3}=\frac{\partial^{\alpha }}{\partial y^{\alpha }}, \end{array}$$

with the property *ℒ*_*i*_[*c*] = 0, where *c* is constant. 

We now define nonlinear operators as

(21) 𝒩1=∂αu∂yα+1σ2∂2u∂x2−2σ4∂v∂t+2λσ4(u3+v2u)−2σ4(∂ϕ∂xu),𝒩2=∂αv∂yα+1σ2∂2v∂x2+2σ4∂u∂t+2λσ4(v3+u2v)−2σ4(∂ϕ∂xv),𝒩3=∂αϕ∂yα−1σ2∂2ϕ∂x2+2λσ2∂(u2+v2)∂x.

The initial guesses are considered as follows:

(22) u0(x,y,t)=u(x,0,t),v0(x,y,t)=v(x,0,t),ϕ0(x,y,t)=ϕ(x,0,t).

In view of the discussion in Section 3, we get the following recursive relations:

(23) u1(x,y,t)=hIyα[ℛ11(u→0,v→0,ϕ→0,x,y,t)],v1(x,y,t)=hIyα[ℛ21(u→0,v→0,ϕ→0,x,y,t)],ϕ1(x,y,t)=hIyα[ℛ31(u→0,v→0,ϕ→0,x,y,t)],

(24) um+1(x,y,t) =um(x,y,t)+hIyα[ℛ1m+1(u→m,v→m,ϕ→m,x,y,t)],vm+1(x,y,t) =vm(x,y,t)+hIyα[ℛ2m+1(u→m,v→m,ϕ→m,x,y,t)],ϕm+1(x,y,t) =ϕm(x,y,t)+hIyα[ℛ3m+1(u→m,v→m,ϕ→m,x,y,t)],                     m=1,2,…,

where

(25) ℛ1m+1(u→m,v→m,ϕ→m,x,y,t) =∂αum∂yα+1σ2∂2um∂x2−2σ4∂vm∂t +2λσ4{(∑j=0muj∑i=0m−juium−j−i)+(∑j=0muj∑i=0m−jvivm−j−i)} −2σ4{∑j=0m∂ϕj∂xum−j},ℛ2m+1(u→m,v→m,ϕ→m,x,y,t) =∂αvm∂yα+1σ2∂2vm∂x2+2σ4∂um∂t +2λσ4{(∑j=0mvj∑i=0m−jvivm−j−i)+(∑j=0mvj∑i=0m−juium−j−i)} −2σ4{∑j=0m∂ϕj∂xvm−j},ℛ3m+1(u→m,v→m,ϕ→m,x,y,t) =∂αϕm∂yα−1σ2∂2ϕm∂x2 +2λσ2{∂∂x((∑j=0mujum−j)+(∑j=0mvjvm−j))}.

## 5. Results Analysis

In this section, some numerical results are presented to support our theatrical analysis. We consider the following initial conditions:

(26) u(x,0,t)=r sech[s(x−ct)]cos⁡⁡[(k1x+k3t)],v(x,0,t)=r sech[s(x−ct)]sin⁡[(k1x+k3t)],ϕ(x,0,t)=f tanh[s(x−ct)],

where

(27) c=k2+σ2k1,r=−(2k3+k12σ2+k22)λ,s=(2k3+k12σ2+k22)σ2,f=(2σ−λ)(1−σ2),

and *k*_*i*_  (*i* = 1,2, 3) are arbitrary constants. 

By the same manipulation as in Section 4, we will have

(28)u1=ℏ[−2frsyαsech⁡[s(−ct+x)]3cos⁡⁡[xk1+tk3]σ4Γ[α+1]   −rs2yαsech[s(−ct+x)]3cos⁡[xk1+tk3]σ2Γ[α+1]   +2r3yαλcos⁡[xk1+tk3]3sech[s(−ct+x)]3σ4Γ[α+1]   +2r3yαλsech[s(−ct+x)]3sin[xk1+tk3]2cos⁡[xk1+tk3]σ4Γ[α+1]   −ryαsech[s(−ct+x)]cos⁡[xk1+tk3]k12σ2Γ[α+1]   −2ryαsech[s(−ct+x)]cos⁡[xk1+tk3]k3σ4Γ[α+1]   −2crsyαsin[xk1+tk3]sech[s(−ct+x)]tanh[s(−ct+x)]σ4Γ[α+1]   +2rsyαsin[xk1+tk3]sech[s(−ct+x)]k1tanh[s(−ct+x)]σ2Γ[α+1]   +rs2yαsech[s(−ct+x)]cos⁡[xk1+tk3]tanh[s(−ct+x)]2Γ[α+1]σ2],v1=ℏ[−2frsyαsech[s(−ct+x)]3sin[xk1+tk3]Γ[α+1]σ4   −rs2yαsech[s(−ct+x)]3sin[xk1+tk3]σ2Γ[α+1]   +2r3yαλcos⁡[xk1+tk3]2sech[s(−ct+x)]3sin[xk1+tk3]σ4Γ[α+1]   +2r3yαλsech[s(−ct+x)]3sin[xk1+tk3]3σ4Γ[α+1]   −ryαsech[s(−ct+x)]sin[xk1+tk3]k12σ2Γ[α+1]   −ryαsech[s(−ct+x)]sin[xk1+tk3]k3σ4Γ[α+1]   +2crsyαcos⁡[xk1+tk3]sech[s(−ct+x)]tanh[s(−ct+x)]σ4Γ[α+1]   −2rsyαcos⁡[xk1+tk3]sech[s(−ct+x)]k1tanh[s(−ct+x)]Γ[α+1]σ2   +rs2yαsech[s(−ct+x)]sin[xk1+tk3]tanh[s(−ct+x)]2Γ[α+1]σ2],(29)ϕ1=2ℏsyα(fs−2r2λ)sech[s(−ct+x)]2tanh[s(−ct+x)]Γ[α+1]σ2.

In the same manner, using recurrence relations in (24) the other components *v*_2_(*x*, *y*, *t*), *v*_3_(*x*, *y*, *t*),…, *u*_2_(*x*, *y*, *t*), *u*_3_(*x*, *y*, *t*),…,  and *ϕ*_2_(*x*, *y*, *t*), *ϕ*_3_(*x*, *y*, *t*),… can be obtained. 

## 6. Convergence and Stability Analysis

This section is devoted to prove the convergence and stability of solutions for fractional initial value problems on a finite interval of the complex axis in spaces of continuous functions.

**Theorem**

If the series *u*_*i*_(*x*, *y*, *t*) = ∑_*m*=0_^*∞*^ *u*_*im*_(*x*, *y*, *t*), *i* = 1,2,…, *n*, converges, where *u*_*im*_(*x*, *y*, *t*) is governed by (15) under the definitions (16), it must be the solution of (7).

**Proof**

Proof is similar to Theorem  3.1 in [17].

Clear conclusion can be drawn from the numerical results and Theorem 6. Our approach provides highly accurate numerical solutions without spatial discretization for the problems. Overall, results show that the proposed approach is unconditionally stable and convergent. In other words, we can always find a proper value of the convergence control parameter *ℏ* to ensure the convergent series solution, and our approximate results agree well with numerical ones. It should be pointed out that the response and stability of this type of problems in general can also be studied in a similar way. For further information see [25]. 

**Figure 1:**
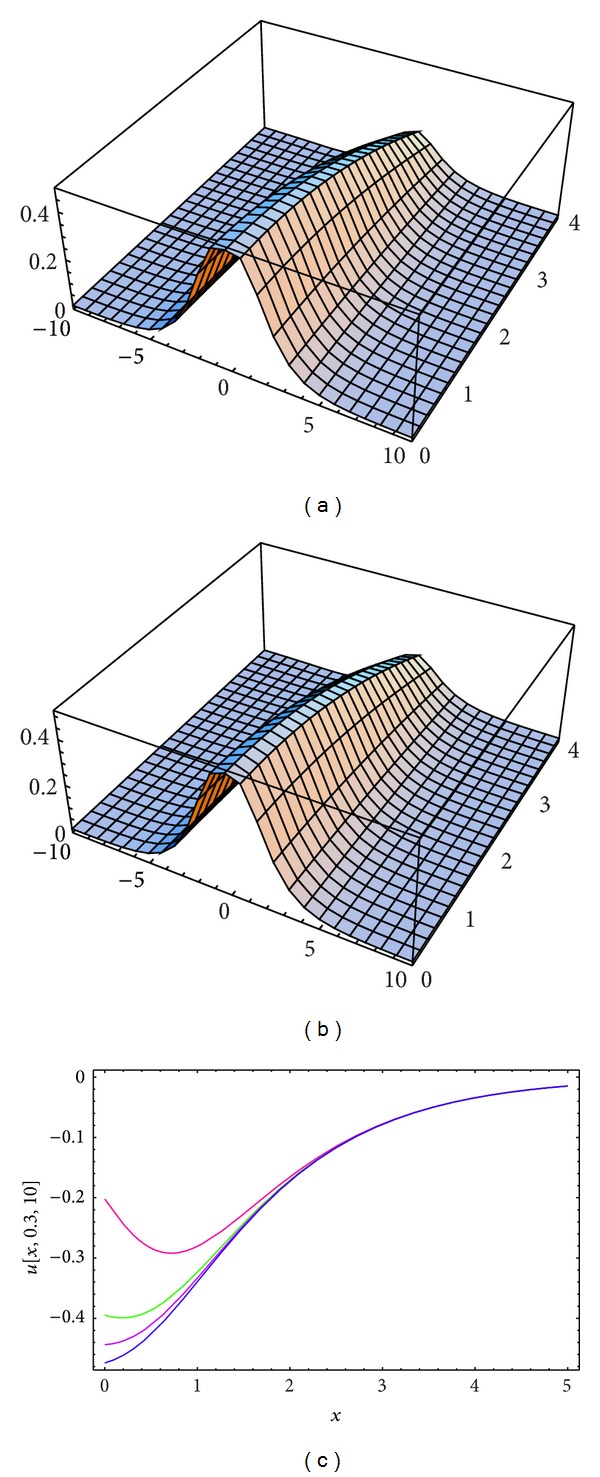
(a) and (b) The surface shows the solution *u*(*x*, *y*, *t*) for (8): (a) approximate solution for *α* = 1.98, *ℏ* = −1.1; (b) exact solution. (c) Four profiles of approximate solutions *u*(*x*, *y*, *t*) for some values of *α*: blue line (*α* = 2), mauve line (*α* = 1.9), green line (*α* = 1.8), and red line (*α* = 1.5), when *k*_1_ = 0.1, *k*_2_ = 0.03, *k*_3_ = −0.3, *σ* = *I*, and *λ* = 1.

**Figure 2:**
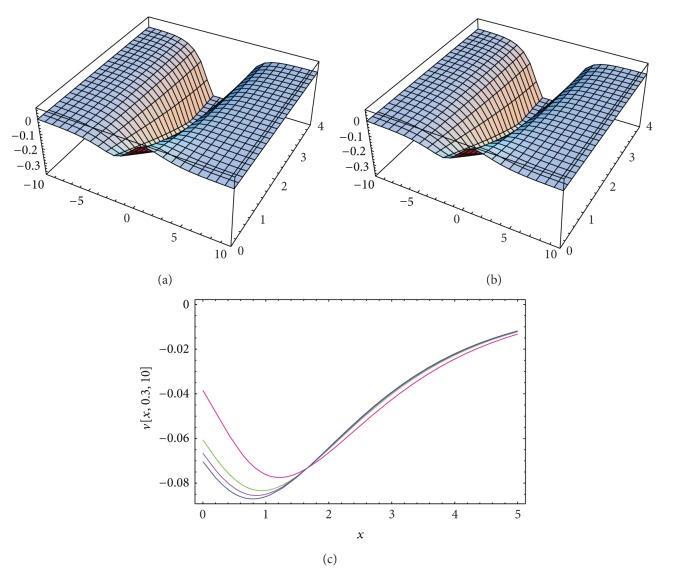
(a) and (b) The surface shows the solution *v*(*x*, *y*, *t*) for (8): (a) approximate solution for *α* = 1.98, *ℏ* = −1.1; (b) exact solution. (c) Four profiles of approximate solutions *v*(*x*, *y*, *t*) for some values of *α*: blue line (*α* = 2), mauve line (*α* = 1.9), green line (*α* = 1.8), and red line (*α* = 1.5), when *k*_1_ = 0.1, *k*_2_ = 0.03, *k*_3_ = −0.3, *σ* = *I*, and *λ* = 1.

**Figure 3:**
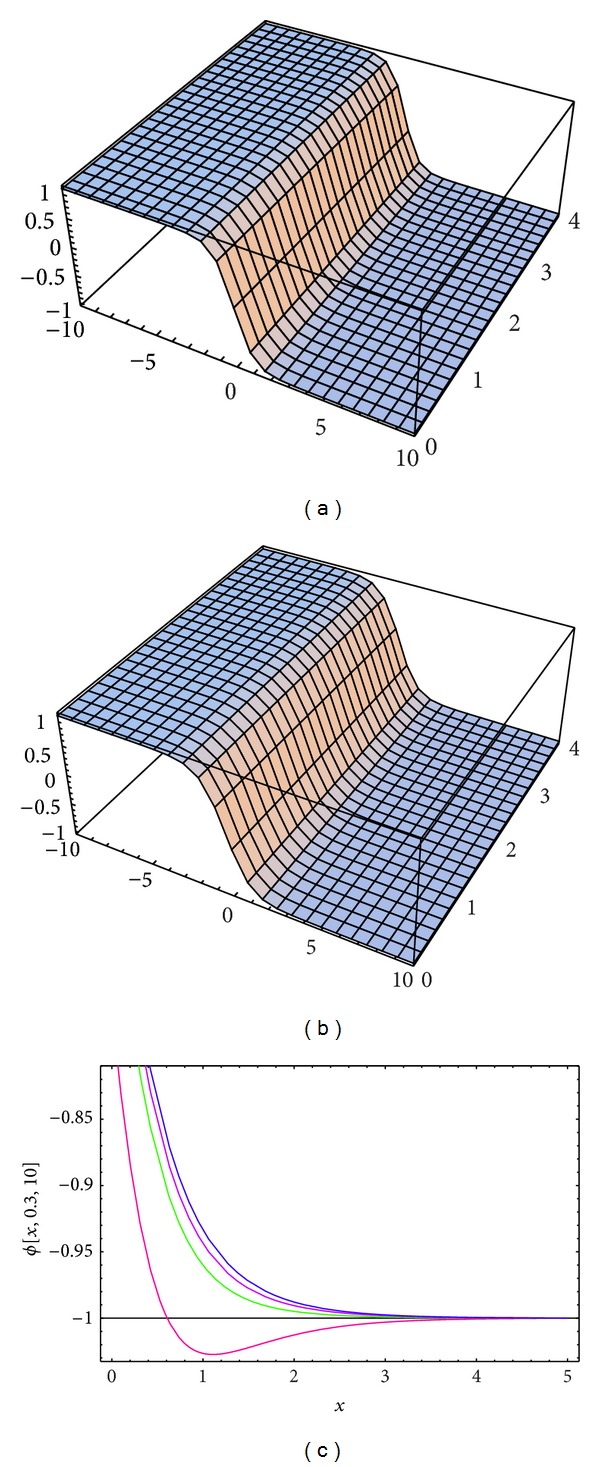
(a) and (b) The surface shows the solution *ϕ*(*x*, *y*, *t*) for (8): (a) approximate solution for *α* = 1.98, *ℏ* = −1.1; (b) exact solution. (c) Four profiles of approximate solutions *ϕ*(*x*, *y*, *t*) for some values of *α*: blue line (*α* = 2), mauve line (*α* = 1.9), green line (*α* = 1.8), and red line (*α* = 1.5), when *k*_1_ = 0.1, *k*_2_ = 0.03, *k*_3_ = −0.3, *σ* = *I*, and *λ* = 1.

Tables 1, 2, and 3 show the absolute errors between the approximate solutions obtained for value of *α* = 1.98 by the homotopy analysis method and the exact solutions. It is to be noted that only the two-order term of the homotopy analysis method solutions for the special case *y* = 0.2, *k*_1_ = 0.1, *k*_2_ = 0.03, *k*_3_ = −0.3, *σ* = *i*, and  *λ* = 1 is used in evaluating the approximate solutions for Tables 1, 2, and 3. Both the exact solutions and the approximate solutions of *u*(*x*, *y*, *t*), *v*(*x*, *y*, *t*), and *ϕ*(*x*, *y*, *t*) (for the same parameters as mentioned before) are plotted in Figures 1, 2, and 3.

## 7. Concluding Remarks

In this paper, the homotopy analysis method has been successfully applied to find the solution of fractional order Davey-Stewartson equations. The convergence and stability of the HAM solution was examined. Results reveal that the solution obtained by the homotopy analysis method is an infinite power series for appropriate initial condition, which can, in turn, be expressed in a closed form, the exact solution. Moreover, in the comparison of HAM with VIM method we will find better approximations. The results show that the homotopy analysis method is a powerful mathematical tool for solving Davey-Stewartson equations of fractional order. In other words, the proposed approach is also a promising method to solve other nonlinear equations. Finally, HAM yields convergent solutions for all values of the relevant parameters whereas a previous study only provided convergent approximate solutions for small *α*. We pointed out that the corresponding analytical and numerical solutions are obtained using Mathematica.

## Figures and Tables

**Table 1:** Absolute errors of *u*(*x*, *y*, *t*).

*x*	*t*					
	0.1			0.5		
	ℏ = −1.1	ℏ = −1	VIM	ℏ = −1.1	ℏ = −1	VIM
20	1.2787 × 10^−8^	1.2831 × 10^−8^	1.284 × 10^−8^	8.3105 × 10^−9^	8.355 × 10^−9^	8.3639 × 10^−9^
17	1.918 × 10^−8^	1.9662 × 10^−8^	1.9759 × 10^−8^	2.602 × 10^−8^	2.6289 × 10^−8^	2.6326 × 10^−8^
14	9.9302 × 10^−7^	1.0008 × 10^−6^	9.9505 × 10^−7^	1.4136 × 10^−6^	1.4301 × 10^−6^	1.4247 × 10^−6^
11	2.1807 × 10^−5^	2.1951 × 10^−5^	2.1948 × 10^−5^	0.2546 × 10^−5^	0.2551 × 10^−5^	2.5473 × 10^−5^

**Table 2:** Absolute errors of *v*(*x*, *y*, *t*).

*x*	*t*					
	0.1			0.5		
	ℏ = −1.1	ℏ = −1	VIM	ℏ = −1.1	ℏ = −1	VIM
20	4.7113 × 10^−8^	4.8836 × 10^−8^	4.7367 × 10^−8^	4.7378 × 10^−8^	4.9292 × 10^−8^	4.7834 × 10^−8^
17	5.1811 × 10^−7^	5.2365 × 10^−8^	5.1823 × 10^−7^	5.0412 × 10^−7^	5.1921 × 10^−7^	5.0445 × 10^−7^
14	5.1731 × 10^−6^	5.2219 × 10^−6^	5.1745 × 10^−6^	4.8430 × 10^−6^	4.8858 × 10^−6^	4.8504 × 10^−6^
11	4.6771 × 10^−5^	4.8037 × 10^−5^	4.6775 × 10^−5^	4.1509 × 10^−5^	4.2922 × 10^−5^	4.1826 × 10^−5^

**Table 3:** Absolute errors of *ϕ*(*x*, *y*, *t*).

*x*	*t*					
	0.1			0.5		
	ℏ = −1.1	ℏ = −1	VIM	ℏ = −1.1	ℏ = −1	VIM
20	4.440 × 10^−16^	8.881 × 10^−16^	9.992 × 10^−16^	4.737 × 10^−16^	6.661 × 10^−16^	9.992 × 10^−16^
17	5.584 × 10^−14^	8.815 × 10^−14^	1.169 × 10^−13^	8.459 × 10^−14^	5.306 × 10^−14^	1.122 × 10^−13^
14	5.956 × 10^−12^	9.577 × 10^−12^	1.269 × 10^−11^	9.205 × 10^−12^	5.655 × 10^−12^	1.219 × 10^−11^
11	6.288 × 10^−10^	1.397 × 10^−9^	1.383 × 10^−9^	1.007 × 10^−9^	5.952 × 10^−10^	1.329 × 10^−9^

## References

[1] Hilfer R (1999). *Applications of Fractional Calculus in Physics*.

[2] Diethelm K, Luchko Y (2004). Numerical solution of linear multi-term initial value problems of fractional order. *Journal of Computational Analysis and Applications*.

[3] Hashim I, Abdulaziz O, Momani S (2009). Homotopy analysis method for fractional IVPs. *Communications in Nonlinear Science and Numerical Simulation*.

[4] Mainardi F, Rionero S, Ruggeeri T (1994). On the initial value problem for the fractional diffusion-wave equation. *Waves and Stability in Continuous Media*.

[5] Momani S, Odibat Z (2008). A novel method for nonlinear fractional partial differential equations: combination of DTM and generalized Taylor’s formula. *Journal of Computational and Applied Mathematics*.

[6] Podlubny I (1999). *Fractional Differential Equations*.

[7] Spasic AM, Lazarevic MP (2005). Electroviscoelasticity of liquid/liquid interfaces: fractional-order model. *Journal of Colloid and Interface Science*.

[8] Daftardar-Gejji V, Bhalekar S (2008). Solving multi-term linear and non-linear diffusion-wave equations of fractional order by Adomian decomposition method. *Applied Mathematics and Computation*.

[9] Daftardar-Gejji V, Jafari H (2007). Solving a multi-order fractional differential equation using adomian decomposition. *Applied Mathematics and Computation*.

[10] Jafari H, Khalique CM, Nazari M (2011). Application of the Laplace decomposition method for solving linear and nonlinear fractional diffusionwave equations. *Applied Mathematics Letters*.

[11] Sweilam NH, Khader MM, Al-Bar RF (2007). Numerical studies for a multi-order fractional differential equation. *Physics Letters*.

[12] Erturk VS, Momani S, Odibat Z (2008). Application of generalized differential transform method to multi-order fractional differential equations. *Communications in Nonlinear Science and Numerical Simulation*.

[13] Liao SJ (1992). *The proposed homotopy analysis technique for the solution of nonlinear problems [Ph.D. thesis]*.

[14] Liao S-J (1995). An approximate solution technique not depending on small parameters: a special example. *International Journal of Non-Linear Mechanics*.

[15] Liao S-J (1997). An approximate solution technique which does not depend upon small parameters—part 2: an application in fluid mechanics. *International Journal of Non-Linear Mechanics*.

[16] Liao S-J (1999). An explicit, totally analytic approximate solution for Blasius’ viscous flow problems. *International Journal of Non-Linear Mechanics*.

[17] Liao SJ (2003). *Beyond Perturbation: Introduction to the Homotopy Analysis Method*.

[18] Jafari H, Seifi S (2009). Homotopy analysis method for solving linear and nonlinear fractional diffusion-wave equation. *Communications in Nonlinear Science and Numerical Simulation*.

[19] Jafari H, Seifi S (2009). Solving a system of nonlinear fractional partial differential equations using homotopy analysis method. *Communications in Nonlinear Science and Numerical Simulation*.

[20] Cang J, Tan Y, Xu H, Liao S-J (2009). Series solutions of non-linear Riccati differential equations with fractional order. *Chaos, Solitons and Fractals*.

[21] Davey A, Stewartson K (1974). On three-dimensional packets of surfaces waves. *Proceedings of the Royal Society of London*.

[22] Luchko YU, Gorenflo R (1999). An operational method for solving fractional differential equations with the Caputo derivatives. *Acta Mathematica Vietnamica*.

[23] Moustafa OL (2003). On the Cauchy problem for some fractional order partial differential equations. *Chaos, Solitons and Fractals*.

[24] Samko G, Kilbas AA, Marichev OI (1993). *Fractional Integrals and Derivatives: Theory and Applications*.

[25] Huang ZL, Jin XL (2009). Response and stability of a SDOF strongly nonlinear stochastic system with light damping modeled by a fractional derivative. *Journal of Sound and Vibration*.

